# A SuperLearner-based pipeline for the development of DNA methylation-derived predictors of phenotypic traits

**DOI:** 10.1371/journal.pcbi.1012768

**Published:** 2025-02-06

**Authors:** Dennis Khodasevich, Nina Holland, Lars van der Laan, Andres Cardenas

**Affiliations:** 1 Department of Epidemiology and Population Health, Stanford University School of Medicine, Palo Alto, California, United States of America; 2 Center for Environmental Research and Community Health (CERCH), University of California Berkeley School of Public Health, Berkeley, California, United States of America; 3 Department of Statistics, University of Washington, Seattle, Washington, United States of America; Children's National Hospital, George Washington University, UNITED STATES OF AMERICA

## Abstract

**Background:**

DNA methylation (DNAm) provides a window to characterize the impacts of environmental exposures and the biological aging process. Epigenetic clocks are often trained on DNAm using penalized regression of CpG sites, but recent evidence suggests potential benefits of training epigenetic predictors on principal components.

**Methodology/findings:**

We developed a pipeline to simultaneously train three epigenetic predictors; a traditional CpG Clock, a PCA Clock, and a SuperLearner PCA Clock (SL PCA). We gathered publicly available DNAm datasets to generate i) a novel childhood epigenetic clock, ii) a reconstructed Hannum adult blood clock, and iii) as a proof of concept, a predictor of polybrominated biphenyl exposure using the three developmental methodologies. We used correlation coefficients and median absolute error to assess fit between predicted and observed measures, as well as agreement between duplicates. The SL PCA clocks improved fit with observed phenotypes relative to the PCA clocks or CpG clocks across several datasets. We found evidence for higher agreement between duplicate samples run on alternate DNAm arrays when using SL PCA clocks relative to traditional methods. Analyses examining associations between relevant exposures and epigenetic age acceleration (EAA) produced more precise effect estimates when using predictions derived from SL PCA clocks.

**Conclusions:**

We introduce a novel method for the development of DNAm-based predictors that combines the improved reliability conferred by training on principal components with advanced ensemble-based machine learning. Coupling SuperLearner with PCA in the predictor development process may be especially relevant for studies with longitudinal designs utilizing multiple array types, as well as for the development of predictors of more complex phenotypic traits.

## Author summary

DNA methylation functions as a vital interface between genes and environment. A wide range of epigenetic predictors have harnessed DNA methylation data to address a variety of research questions including improving our understanding of the biological aging process and characterizing past exposure to environmental toxins. However, the methodology used to develop most existing epigenetic predictors is subject to several limitations including the influence of technical variables, batch effects, and difficulty modeling complex relationships between the variable of interest and DNA methylation. Here, we introduce a novel method for the development of epigenetic predictors that combines the improved reliability conferred by training on principal components with advanced ensemble-based machine learning. We demonstrate the potential benefits of this novel procedure by developing a novel childhood epigenetic clock, reconstructing the Hannum clock, and producing a predictor of polybrominated biphenyl exposure. This novel training methodology may be especially relevant for the development of epigenetic predictors of complex phenotypic traits, which often suffer from poor performance using the traditional development methodology, and for the improvement of the reliability of epigenetic clocks for studies with longitudinal designs utilizing multiple array types.

## Introduction

DNA methylation functions as a vital interface between genes and environment, which might serve as a sensitive and stable indicator of past exposures [1]. Often, research on DNA methylation has focused on characterizing differentially methylated CpG sites and regions throughout the genome in response to environmental exposures or disease status. However, the proliferation of standardized DNA methylation microarrays has promoted the study of DNA methylation beyond the context of gene expression with the usage of DNA methylation-derived predictors of phenotypic traits. DNA methylation data has been used to develop several predictors of phenotypic traits including age [2,3], smoking status [4], and various clinical outcomes [5]. In particular, Horvath’s pan-tissue clock and other epigenetic clocks highlight the utility of DNA methylation-based phenotypic predictors through their ability to both generate accurate chronological age estimates, as well as provide insights into the influence of various exposures on the biological aging process and subsequent risk for morbidity and mortality measures [6]. The introduction of Horvath’s epigenetic clock has since spawned significant research interest in epigenetic aging and a myriad of epigenetic clocks have been subsequently introduced including gestational age predictors [7], phenotypic age predictors like PhenoAge [8] and GrimAge [9], and biomarkers capturing the longitudinal pace of aging [10].

Although epigenetic clocks and other DNA methylation-based phenotypic predictors vary widely in terms of purpose and efficacy, they often share a common development methodology: elastic net regression. Elastic net regression is a form of penalized regression that combines LASSO and ridge regression penalties, resulting in a subset of variables from a high dimensional dataset that are most predictive of the outcome of interest [11]. This procedure results in an easily interpretable output of a small subset of CpG sites and their associated beta coefficients which can then be linearly combined with the observed DNA methylation values to generate an individual prediction.

DNA methylation is commonly measured with Illumina microarrays, for example the 450K and EPIC microarrays, which provide highly reproducible means of characterizing DNA methylation across the epigenome. However, the reliability of individual CpG sites can be influenced by several factors including batch effects [12], variability within individual sites [13], and array type differences [14,15]. This limited reliability of individual CpG sites can become a key issue for epigenetic clocks and other DNA methylation-derived predictors which often use only a small subset of CpG sites, usually ranging from a few dozen to a few hundred, to predict a trait. Recently, Higgins-Chen and colleagues proposed training epigenetic clocks on the principal components generated from CpG-level data to overcome the limitations of relying on individual CpG sites [16]. The PCA clocks were found to improve agreement between replicate samples, improve the detection of clock associations, and better enable the study of longitudinal trajectories of epigenetic aging compared to CpG-trained clocks [16]. The substantial data reduction conferred by the principal component analysis of DNA methylation data may also enable the implementation of more advanced machine learning methodology. One popular modern machine learning methodology is SuperLearner, which is an algorithm that uses cross-validation to evaluate the performance of multiple candidate algorithms and create an ensemble model consisting of a weighted combination of the individual candidate algorithms [17]. Given sufficient data, this process will, at worst, produce a model equivalent to the best performing individual model and, at best, improve upon all individual models by creating an optimally weighted ensemble model [17].

DNA methylation-based predictors have emerged as key biomarkers for environmental health research, and it is vital that these predictors maximize the signal-to-noise ratio and are capable of modeling complex exposure-response relationships. Leveraging these methodological advances, we developed a pipeline capable of simultaneously training DNA methylation-based predictors using 3 approaches integrating established methods; 1) the traditional approach based on elastic net regression of the CpG matrix, 2) elastic net regression of the principal component matrix, and 3) the ensemble prediction derived from running a SuperLearner model on the principal component matrix. We hypothesized that coupling the principal component training method with SuperLearner would lead to improved predictions compared to the CpG-based and standard PCA-based clock training methods. To test this hypothesis, we compare the 3 clock development methodologies with the training and testing of a novel childhood clock, the traditional Hannum clock, and a predictor of polybrominated biphenyl (PBB) exposure.

## Results

### Childhood clock

**Fig 1:**
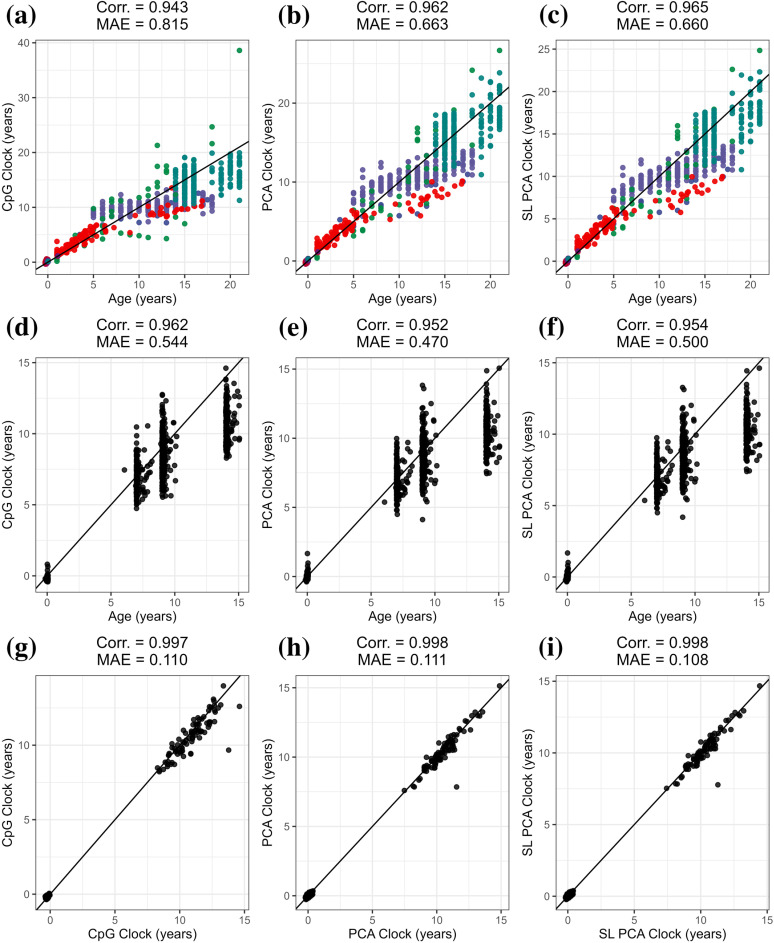
Childhood clock primary summary. Correlation coefficients and median absolute error (MAE) to chronological age for each childhood clock development method for the childhood training data for the traditional CpG clock (a), the PCA clock (b), and the SL PCA clock (c), CHAMACOS testing data (d–f), and agreement between alternate array replicates in CHAMACOS (g–i). Training data color corresponds to GEO dataset. The 1:1 line is shown in black.

Three novel childhood clocks applicable to cord blood, buccal samples, and peripheral blood samples in children ranging from birth to 21 years of age were developed using each of the clock development methods with a collection of 859 publicly available datasets. On the training dataset, the PCA clock resulted in an improvement in correlation and median absolute error (MAE) with chronological age (Corr. = 0.962, MAE = 0.663) compared to the CpG clock (Corr. = 0.943, MAE = 0.815), and the SL PCA clock achieved the highest correlation and lowest MAE (Corr. = 0.965, MAE = 0.660) (Fig 1A–C). As our primary testing dataset, we harnessed 976 DNA methylation measures obtained from children ranging from birth to 14 years of age from the CHAMACOS cohort, obtained from the 450K array at birth and age 9 years and the EPIC array at age 7 and 14 years. The implementation of the PCA clock (Corr. = 0.952, MAE = 0.470) resulted in a small decrease in correlation and decrease in MAE relative to the CpG clock (Corr. = 0.962, MAE = 0.544) (Fig 1D–F). The SL PCA clock then modestly improved the correlation to chronological age relative to the PCA clock (Corr. = 0.954, MAE = 0.500). Bootstrapping also indicated that the CpG clock significantly outperformed the PCA clock (95% CI: 0.005 – 0.014) and SL PCA clock (95% CI: 0.003 – 0.012) in terms of correlation, the SL PCA clock outperformed the standard PCA clock (95% CI: 0.001 – 0.002) in terms of correlation, while the MAE did not significantly differ between any of the predictors. Furthermore, when comparing the novel childhood clocks to several commonly used external epigenetic clocks, the Horvath and PC Horvath outperformed the childhood clocks in terms of correlation, while the novel childhood clocks outperformed each of the external clocks in terms of MAE by a large margin (**Table B in** **S1 Text**). In addition to the primary DNAm array measurements in the CHAMACOS cohort, a subset of 193 participants possessed replicate measurements run on the alternate array, totaling to 108 cord blood and 85 samples at 14 years of age having DNAm measures on both 450K and EPIC arrays, allowing for the evaluation of array-based differences in age predictions. All three clocks produced relatively high agreement between replicates run on alternate arrays, with the SL PCA clock achieving the highest correlation and lowest MAE (Corr. = 0.998, MAE = 0.108) (Fig 1G–I). Bootstrapping also indicated that the SL PCA clock significantly outperformed the PCA clock in terms of correlation (95% CI: 2.13e-05 - 2.62e-04), while none of the other differences in correlation or MAE were significant. Additionally, the SL PCA predictor achieved the lowest MAE for comparisons between replicate samples in comparison to the external epigenetic clocks as well (**Table B in** **S1 Text**).

We additionally examined the three clocks in the CHAMACOS dataset considering cord blood and childhood blood samples separately. The PCA clock (Corr. = 0.342, MAE = 0.095) and SL PCA clock (Corr. = 0.339, MAE = 0.105) display substantially higher correlation and lower MAE with gestational age at birth compared to the CpG clock (Corr. = 0.135, MAE = 0.248) (**Fig A in** **S1 Text**). Low correlation with gestational age at birth also appeared with the age predictions derived from the Horvath panTissue clock (Corr. = 0.108, MAE = 1.445) and PC Horvath clock (Corr. = 0.212, MAE = 1.746) despite their training samples containing cord blood samples. (**Table B in** **S1 Text**) When considering the childhood sample, the CpG clock displayed the highest correlation with age (Corr. = 0.756, MAE = 1.444), with the PCA clock (Corr. = 0.691, MAE = 1.345) and SL PCA clock (Corr. = 0.702, MAE = 1.388) both displaying lower MAE compared to the CpG clock (**Fig A in** **S1 Text**). The slight drop in correlation with the PCA clock is also partially counteracted with the SL PCA clock.

The CHAMACOS cohort dataset also provides the opportunity to test the three clocks in a longitudinal analysis involving exposures previously shown to be associated with epigenetic age acceleration in children, namely prenatal phthalate exposure. All three clocks produced similar effect estimates for the associations between prenatal phthalate exposure and cell-adjusted epigenetic age acceleration throughout childhood in the GEE models (**Fig B in** **S1 Text**). However, the average width of the 95% confidence intervals tended to decrease with the PCA clock and the SL PCA clocks, with the SL PCA clock producing the narrowest average 95% confidence intervals, indicating higher precision of the estimates derived from PCA and SL PCA clocks (**Fig C in** **S1 Text**). Additionally, the external epigenetic clocks exhibited markedly wider confidence intervals compared to the novel childhood clocks (**Figs D–E in** **S1 Text**).

Lastly, we tested the childhood clocks on a sample of 127 children ranging from 4 days old to 17 years old, consisting of 43 Multisystem Inflammatory Syndrome in Children patients, 15 COVID-19 cases, and 69 healthy controls. The implementation of the PCA clock (Corr. = 0.892, MAE = 1.875) resulted in a slight drop in correlation and increase in MAE relative to the CpG clock (Corr. = 0.914, MAE = 1.122) (**Fig A in** **S1 Text**). The SL PCA clock (Corr. = 0.895, MAE = 1.861) then improved both the correlation and MAE relative to the standard PCA clock. Bootstrapping also indicated that the CpG clock significantly outperformed the PCA clock (95% CI: 0.001 – 0.044) and the SL PCA clock outperformed the standard PCA clock (95% CI: 0.002 – 0.004) in terms of correlation, while the CpG clock significantly outperformed the PCA clock (95% CI: -1.043 - -0.389) and SL PCA clock (95% CI: -1.031 - -0.377) in terms of MAE. Additionally, each of the childhood clocks outperformed the external clocks in terms of MAE by a large margin in the GSE193879 sample, while the PC Horvath clock achieved the highest correlation. (**Table B in** **S1 Text**).

### Hannum clock

**Fig 2:**
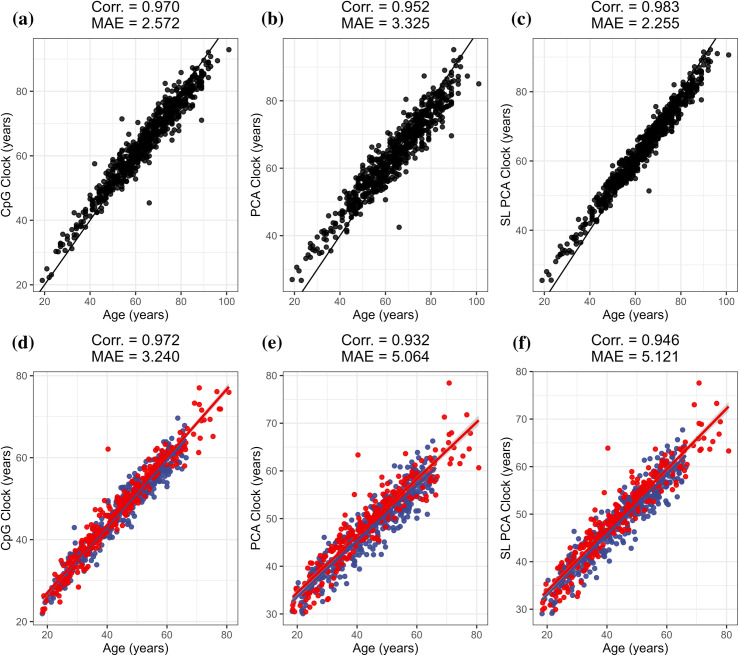
Adult clock primary summary. Correlation coefficients and median absolute error (MAE) to chronological age for each Hannum clock development method for the Hannum training data for the traditional CpG clock (a), the PCA clock (b), and the SL PCA clock (c), with the 1:1 line is shown in black. Correlation coefficients and MAE for the GSE84727 testing data (d–f) with color corresponding to schizophrenia case status, with red indicating schizophrenia case status and blue indicating a control.

We next sought out to compare the three clock development methodologies using the original Hannum clock (GSE40279) training dataset of 656 adult samples, creating a Hannum CpG clock which roughly approximates the original Hannum clock, a Hannum PCA clock, and a Hannum SL PCA clock. In the training dataset, the implementation of the PCA clock (Corr. = 0.952, MAE = 3.325) resulted in a drop in correlation and increase in MAE relative to the CpG clock (Corr. = 0.970, MAE = 2.572), which was then reversed with the SL PCA clock (Corr. = 0.983, MAE = 2.255) to produce the highest correlation and lowest MAE (Fig 2A–C). We tested the Hannum clocks on the GSE84727 dataset consisting of 665 schizophrenia cases and controls ranging in age from 18.3 to 80.7 years old. The PCA clock (Corr. = 0.932, MAE = 5.064) and SL PCA clock (Corr. = 0.946, MAE = 5.121) both resulted in decreased correlation and increased the MAE relative to the CpG clock (Corr. = 0.972, MAE = 3.240), with the SL PCA clock improving the correlation relative to the PCA clock (Fig 2D–F). Bootstrapping also indicated that the CpG clock significantly outperformed the PCA clock (95% CI: 0.032 – 0.047) and SL PCA clock (95% CI: 0.020 – 0.031) in terms of correlation, the SL PCA clock outperformed the standard PCA clock (95% CI: 0.011 – 0.017) in terms of correlation, and the MAE for the CpG clock was significantly lower than the PCA clock (95% CI: -2.230 – -1.499) and the SL PCA clock (95% CI: -2.239 – -1.494). We further compared performance of the three adult clocks to several commonly used epigenetic clocks in the GSE84727 dataset. The CpG clock and SL PCA clock both outperformed each external clock in terms of correlation, while the CpG clock featured the lowest observed MAE. (**Table B in** **S1 Text**) Similar to our findings with the CpG and PC Hannum clocks, the PC Horvath clock exhibited modestly decreased correlation and increased MAE relative to the standard Horvath clock. This testing dataset further allowed for testing the association between schizophrenia case status and epigenetic age acceleration. The PCA clock (Beta = 1.14, SE = 0.23, p = 9.7e-07) and SL PCA clock (Beta = 0.97, SE = 0.22, p = 1.2e-05) both produced more significant effect estimates compared to the CpG clock (Beta = 0.51, SE = 0.20, p = 0.012), with the SL PCA clock producing an effect estimate with a smaller standard error compared to the PCA clock. These findings suggest that, despite the decreased correlation and increased MAE with chronological age observed with the PCA and SL PCA clocks within this testing dataset, these clocks may still be capturing relevant inter-individual differences in biological aging, consistent with the noise reduction hypothesis. The PhenoAge (Beta = 1.70, SE = 0.41, p = 4.1e-05), PC PhenoAge (Beta = 2.27, SE = 0.35, p = 1.3e-10), and PC GrimAge clocks (Beta = 3.33, SE = 0.29, p <2e-16) all produced higher magnitude effect estimates compared to the three versions of the Hannum clock, which is not surprising given that each of these clocks were trained to predict specific aspects of biological aging. However, each of the three adult clocks produced lower standard errors compared to the external clocks.

We next used the GSE55763 testing dataset consisting of 11 duplicate samples to characterize the reliability of the three versions of the Hannum clock. The CpG clock (Corr. = 0.989, MAE = 1.250) displayed substantially higher MAE compared to both the PCA clock (Corr. = 0.997, MAE = 0.285) and the SL PCA clock (Corr. = 0.997, MAE = 0.422), with the PCA clock achieving the lowest MAE and highest correlation. The PC and SL PCA clocks performed similarly well to the external PC-based clocks, with the PC Horvath and PC GrimAge clocks achieving the lowest MAE among the external clocks and only the PC GrimAge and PC PhenoAge clocks achieving higher correlations than the SL PCA clock. (**Table B in** **S1 Text**) Finally, we used the GSE174422 testing dataset consisting of 128 duplicate samples from the Sister’s Study to test the reliability of the three versions of the Hannum clock. For both sets of replicates, the PCA clock (Corr._1_ = 0.816, MAE_1_ = 3.585) (Corr._2_ = 0.820, MAE_2_ = 3.642) and SL PCA clock (Corr._1_ = 0.833, MAE_1_ = 3.660) (Corr._2_ = 0.835, MAE_2_ = 3.725) reduced the correlation and increased the MAE compared to the CpG clock (Corr._1_ = 0.920, MAE_1_ = 2.605) (Corr._2_ = 0.917, MAE_2_ = 2.583), with the SL PCA clock improving the correlation relative to the standard PCA clock (**Fig F in** **S1 Text**). All clocks produce relatively high agreement between duplicates, with the CpG clock (Corr. = 0.983, MAE = 0.902) achieving the lowest MAE and the PCA clock (Corr. = 0.985, MAE = 0.999) achieving the highest correlation (**Fig F in** **S1 Text**). Bootstrapping also indicated that the PCA clock significantly outperformed the SL PCA clock in terms of correlation between duplicates (95% CI: -0.006 – -0.001), while none of the other differences in correlation or MAE were significant. All three versions of the Hannum clock also outperformed the Horvath and PhenoAge clocks within this context, performed similarly well to the PC Horvath and PC PhenoAge clocks in terms of correlation, while the PC PhenoAge and PC GrimAge clocks achieved the lowest MAE (**Table B in** **S1 Text**).

### PBB predictor

**Fig 3:**
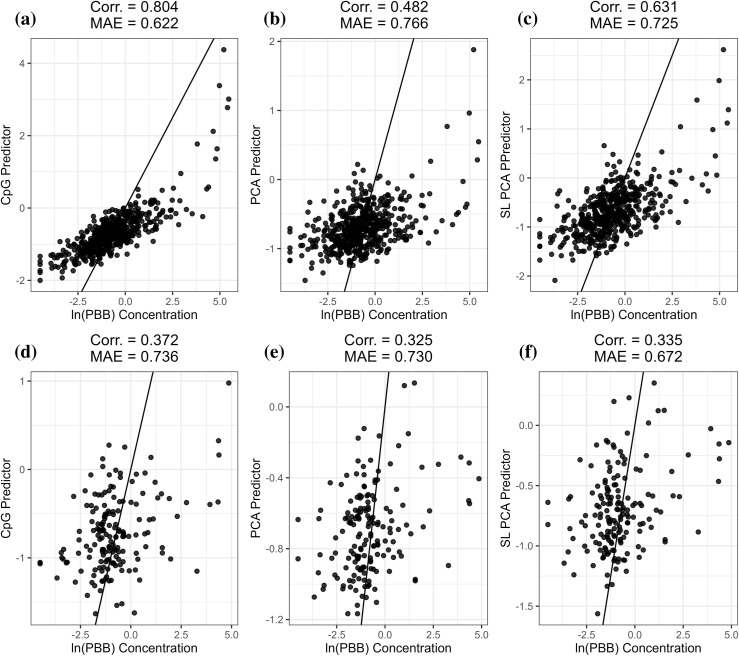
Correlation coefficients and median absolute error (MAE) to observed log-transformed PBB concentrations for the training data with the traditional CpG predictor (a), the PCA predictor (b), and the SL PCA predictor (c), and the testing data (d–f). The 1:1 line is shown in black.

As proof of principal for biomarker development of more complex exposures, we then utilized the GSE116339 dataset consisting of 673 adults from the Michigan PBB registry to develop a predictor of serum polybrominated biphenyl (PBB) exposure. We randomly split the dataset into a training sample of 505 individuals and a testing sample of 168 individuals. Within the training dataset, the CpG predictor (Corr. = 0.804, MAE = 0.622) achieved the highest correlation and lowest MAE, with the SL PCA predictor (Corr. = 0.631, MAE = 0.725) improving both the correlation and MAE relative to the PCA predictor (Corr. = 0.482, MAE = 0.766) (Fig 3A–C). Within the testing dataset, the PCA predictor (Corr. = 0.325, MAE = 0.730) resulted in decreased correlation and decreased MAE relative to the CpG predictor (Corr. = 0.372, MAE = 0.736) with the SL PCA predictor (Corr. = 0.335, MAE = 0.672) improving the correlation relative to the PCA predictor and achieving the lowest overall MAE (Fig 3D–F). Bootstrapping also indicated that none of the differences in correlation or MAE between the three predictors differed significantly.

To examine the robustness of these findings against differential training/testing data splits, we repeated the training process with 10 random training/testing data splits, then averaged correlation and MAE measures across all 10 permutations. Across the training datasets, the CpG predictor exhibited the highest average correlation and lowest average MAE (Corr. = 0.819, MAE = 0.606), with the SL PCA predictor (Corr. = 0.623, MAE = 0.691) improving both the average correlation and MAE relative to the PCA predictor (Corr. = 0.559, MAE = 0.718). Across the testing datasets, the CpG predictor exhibited the highest average correlation (Corr. = 0.422, MAE = 0.764). Both the PCA predictor (Corr. = 0.335, MAE = 0.758) and SL PCA predictor (Corr. = 0.322, MAE = 0.757) exhibited lower average MAE compared to the CpG predictor, with the SL PCA predictor achieving the lowest average MAE.

## Discussion

We developed a bioinformatic pipeline capable of efficiently training DNA methylation-derived predictors of phenotypic traits using three methodologies: a CpG-based predictor based on elastic net regression of a CpG matrix, a PCA-based predictor based on elastic net regression of a principal component matrix calculated from a CpG matrix, and a SuperLearner ensemble predictor based on the principal component matrix. We then tested the utility of predictors derived from each of the three methods with the development of a novel childhood epigenetic clock, a revisiting of the Hannum epigenetic clock, and the development of a novel predictor of PBB exposure. We found evidence that the SL PCA predictor development approach could help to maintain the noise reduction benefits conferred by training predictors on principal components, while allowing for the incorporation of more advanced machine learning algorithms to further improve prediction performance.

Although epigenetic clocks are a valuable biomarker for understanding the determinants of biological aging in human populations, technical noise surrounding epigenetic age predictions remains a pivotal issue [16]. Epigenetic clocks have traditionally been trained on individual CpG sites, the reliability of which can be influenced by several factors including batch effects [12], variability within individual sites [13], and array type differences [14,15]. Several strategies have been proposed to improve the reliability of epigenetic clocks, including increasing training sample data size [18], a noise barometer approach [19], and stability selection of CpG sites [20]. Recently, Higgins-Chen and colleagues proposed an alternative approach of training epigenetic clocks on the principal components generated from CpG-level data to overcome the limitations of relying on individual CpG sites [16]. They found that PCA-trained clocks increased agreement between replicate samples and improved ability to detect clock associations. This improved reliability is likely due to the separation of noise from age-related signals and the pooling of information from a large number of CpG sites as a result of applying principal component analysis. Building upon the improved reliability conferred by training predictors on principal components and substantial data reduction provided by the principal component analysis of DNA methylation data, we developed an efficient pipeline for the creation of ensemble epigenetic predictors using SuperLearner, an algorithm that uses cross validation to evaluate the performance of multiple preselected candidate learning models and create an ensemble model consisting of an optimally weighted combination of the individual candidate models [17]. The primary advantage of utilizing SuperLearner in the context of training epigenetic predictors is the ability to include models that allow for nonlinear relationships, interactions between features, and penalized regression models with various penalty terms, rather than relying on a single model. SuperLearner then uses cross-validation to assess the performance of each included algorithm, then creating an optimally weighted combination to generate a final prediction. The SuperLearner methodology represents a realistic scenario where investigators may suggest the use of alternate algorithms or algorithms with modified parameters, while providing a principled way to evaluate the performance of various input algorithms. Previously, SuperLearner has been sparingly applied to epigenetic predictor development, with only one study constructing a sperm epigenetic clock using a subset of age-associated CpG sites [21].

We developed a novel childhood clock applicable to children from birth to 21 years of age on samples derived from cord blood, peripheral blood, and buccal cells using each of the three development methodologies. These three sample types represent the most common biological samples gathered in pediatric studies, so this clock provides a valuable child multi-tissue aging marker. Second generation epigenetic clocks, like GrimAge and PhenoAge, have largely focused on adult populations. Understanding the determinants and consequences of epigenetic aging in pediatric populations is becoming increasingly important, so it is vital to have highly reliable epigenetic clocks applicable across the entire pediatric age range in common tissue types used in pediatric populations [22].

We found that both the PCA Clock and SL PCA Clock improved agreement between replicates run on 450K and EPIC arrays in the CHAMACOS cohort compared to both the CpG Clock and several external epigenetic clocks. Although different versions of DNAm arrays have high overall agreement, individual CpG sites may be prone to substantial array-based differences in methylation [14,15]. CpG-based predictors rely on a relatively small number of CpG sites, whereas PCA and SL PCA predictors utilize a large scale pattern spread across a larger number of CpG sites, potentially limiting the influence of array-based differences. This higher agreement in predictions between replicate samples run on different arrays is becoming increasingly important for ongoing cohort studies making use of DNAm data obtained from various arrays. Furthermore, when applied to cord blood samples, we found that both the PCA Clock and SL PCA Clock exhibited substantially higher correlations with gestational age at birth compared to the CpG Clock and each external epigenetic clock. The low applicability of CpG-derived multi-tissue clocks in cord blood may hinder the ability to do analysis of epigenetic aging in cord blood samples using these measures. When applied to a longitudinal analysis of prenatal phthalate exposure, all three clocks exhibited similar effect estimates but with varying levels of precision, with the SL PCA Clock achieving the narrowest average confidence interval width. This improved precision under the same sample size when using the SL PCA Clock may be especially relevant for studies with limited power due to small sample size or technical variance.

We additionally revisited the original Hannum epigenetic clock, creating three adult clocks to compare the different developmental methods. The Hannum clock was among the first epigenetic clocks and continues to be routinely used to examine the impacts of exposures on biological aging [3,6]. As with some of the childhood clock testing datasets, we found a reoccurring trend of the PCA Clocks decreasing correlation and increasing MAE compared the CpG Clocks, with the implementation of the SL PCA Clocks then improving correlation and/or MAE compared the standard PCA Clocks. Interestingly, despite frequently observing lower correlation and higher MAE with chronological age with the PCA and SL PCA Clocks, we still found high reliability of epigenetic age predictions between duplicate samples and improved ability to detect relevant biological associations. We observed stronger positive estimates for the association between schizophrenia case status and epigenetic age acceleration with the PCA and SL PCA clocks relative to the traditional CpG clock. Schizophrenia case status has previously been found to be associated with epigenetic age acceleration, with stronger associations observed when using second generation epigenetic clocks [23]. These results may indicate that training epigenetic clocks on principal components, rather than individual CpG sites, may result in an improved signal-to-noise ratio.

Lastly, we developed a novel predictor of polybrominated biphenyl (PBB) exposure, finding the lowest MAE in the testing dataset with the SL PCA predictor. A long-standing hypothesis is that DNA methylation functions as a vital interface between genes and environment, serving as a sensitive and stable indicator of past exposures [1]. Several studies have developed DNAm-derived predictors of other phenotypic traits including plasma protein levels [9], smoking status [4], and various clinical outcomes [5], all utilizing a similar methodology revolving around elastic net regression of a CpG matrix. In addition to the potential noise reduction benefits of training predictors on principal components, the data reduction conferred by this step enables the use of more advanced machine learning methodologies to the predictor development process, allowing for the incorporation of nonlinear relationships and interaction terms. It is important to note that the correlations for our PBB predictor ranged from 0.33 to 0.37 in the testing datasets. However, this may have important implications for the development of future DNAm-based predictors of phenotypic traits, which tend to exhibit more complex relationships with DNA methylation and often suffer from relatively low correlations. For example, the correlations between observed biomarker levels and GrimAge component DNAm-based surrogate biomarker predictions ranged from 0.35 to 0.66 in the reported testing datasets, indicating room for improvement in the development of similar predictors [9].

Our findings subject to a few relevant limitations. First, both the PCA and SL PCA clock development processes represent a non-trivial increase in computational requirements compared to the traditional CpG-based clock development, both for the training and testing components. However, we argue that the noise-reduction provided by the PCA- and SL PCA-based methods may outweigh this increased computational burden and advances in modern computing readily enable these increased computational requirements. Training epigenetic predictors on principal components also limits the interpretability of the individual CpG sites contributing to the predictions. CpG-based clocks consist of a small number of CpG sites, and it is common to characterize the locations of these sites to determine what genes and biological pathways are represented. The principal component-based methods sacrifice some of this simplicity by pooling larger patterns spread across a large number of CpG sites. However, if the goal is biomarker development, the biological interpretation of component CpG sites becomes less relevant. Further interpretation of the input principal components is still possible through characterization of the loadings assigned to CpG sites if desired. The implementation of SuperLearner and the final ensemble prediction then adds another layer of complexity. However, the SuperLearner ensemble predictor can be intuitively explained as a combination of individual predictions obtained from various algorithms. Additionally, since our goal was to provide a tractable computational pipeline using SuperLearner specifically, we did not systematically compare our SuperLearner-based method other established ensemble machine learning approaches such as gradient boosting or random forests. Relatedly, SuperLearner provides the ability to consider a wide variety of learners, variable screening methods, and tuning of model hyperparameters, and our work only considered a fraction of all available algorithms. Future work can take advantage of the provided pipeline to examine the influence of each of these parameters on the epigenetic predictor development process. Finally, the differences in the performance of predictions derived from each of the three development methods often only represented incremental changes. However, in the analyses presented here, we found evidence for stronger effect estimates with narrower confidence intervals when using epigenetic age acceleration measures derived from the PCA and SL PCA clocks, despite the incremental differences in the predictions, suggesting the capture of more biologically relevant signals. Furthermore, any small improvements in the precision of DNAm-based predictions are likely to have a beneficial impact considering the small sample sizes typically found in epigenetic studies. The benefits of the PCA and SL PCA training procedures may also be most prominent in longitudinal settings and datasets containing DNA methylation measurements obtained from multiple arrays, a scenario that is likely to arise in ongoing cohort studies.

Our study also features several key strengths. We were able to compare the CpG, PCA, and SL PCA methods for developing DNAm-derived predictors of phenotypic traits across a wide range of testing scenarios in three distinct contexts: the development of a novel childhood epigenetic clock, revisiting of the traditional Hannum epigenetic clock, and a predictor of environmental PBB exposure. The novel childhood epigenetic clock may aid in the study of epigenetic aging in pediatric populations across commonly collected tissue types, with benefits that may be especially relevant for longitudinal cohorts containing DNA methylation measures of multiple tissue types and array types. Finally, we provide the code underlying the pipeline presented in this study to allow researchers to efficiently develop and compare DNAm-derived predictors of phenotypic traits using all three of the CpG, PCA, and SL PCA based methods in additional contexts.

## Methods

### Ethics statement

The University of California, Berkeley Committee for the Protection of Human Subjects approved all study activities for the CHAMACOS cohort. Written, informed consent was obtained from all participating mothers at all study visits, child verbal assent was obtained starting at age 7 years, child written assent was obtained starting at age 12 years, and child written consent was obtained at age 18 years. Expanded information on working with CHAMACOS cohort data is available on the Center for Environmental Research and Community Health website (https://cerch.berkeley.edu/investigators) [24].

### Epigenetic predictor development pipeline

We developed a pipeline to simultaneously train three epigenetic predictors from the same DNA methylation dataset; i) a CpG predictor, ii) a PCA predictor, and iii) a SuperLearner PCA predictor. A schematic representation of the overall training procedure is provided in **Fig G in** S1 Text.

#### CpG predictor construction.

A cross-validated elastic net regression model with 10 folds is trained on the DNA methylation beta matrix to predict the phenotypic variable of interest (e.g., chronological age or an environmental exposure) using the R package *biglasso* [25]. The beta coefficient matrix from selected probes is then saved and defines the CpG Predictor. Generating predictions from the CpG Predictor is accomplished by calculating a simple linear combination of the beta coefficients with selected DNA methylation values in the testing dataset.

#### PCA predictor construction.

The principal component clock construction follows the procedure set forth by Higgins-Chen et al. [16]. Briefly, principal component analysis is performed on the DNA methylation beta matrix using the R *prcomp* function, forming a principal component matrix with as many principal components as samples in the training dataset. Low-variance principal components are trimmed, then a cross-validated elastic net regression model with 10 folds is trained on the principal component matrix to predict the phenotypic variable of interest using the *cv.glmnet* R function. Beta coefficients for nonzero principal components identified in the elastic net regression and instructions for projecting the DNA methylation beta matrix onto the principal component matrix are saved and presented as the PCA Predictor. Generating predictions from the PCA Predictor is accomplished by projecting testing data onto the principal components using the centering from the original training data, followed by calculating the linear combination of the beta coefficients with the principal component values in the testing dataset.

#### SuperLearner PCA predictor construction.

First, principal component analysis is performed on the DNA methylation beta matrix using the R *prcomp* function. The full principal component matrix is then used as the X-value input for a SuperLearner model with 10-fold cross-validation using the *SuperLearner* R package [26]. SuperLearner is an algorithm that uses cross-validation to compare the performance of multiple input models and generate an ensemble predictor formed as the optimal weighted combination of the input algorithms. Input algorithms can include elastic net regression, ridge regression, LASSO regression, random forest, as well as several other more data-adaptive algorithms and screening algorithms. Algorithm library specification should be tailored to the specific research question at hand and characteristics of the training datasets, and an informative guide to specifying a SuperLearner is provided by Phillips et al. [27]. The instructions for projecting the DNA methylation beta matrix onto the principal component matrix and the SuperLearner model are saved and presented as the SL PCA Predictor. Generating predictions from the SL PCA Predictor is accomplished by projecting testing data onto the principal components from the original training data, using the predicted principal components as inputs for prediction functions for each of the included SuperLearner algorithms, then generating an ensemble prediction as the optimal weighted combination of these individual predictions based on the original training data. All SuperLearner libraries used to train each of the predictors, as well as the final weights given to each algorithm, in this study are presented in the **Table A in** **S1 Text**. Generally, we found that including several penalized regression algorithms with varying combinations of lasso and ridge penalties were sufficient for predictions of chronological age, while predictors of more complex traits, such as environmental exposures benefited from the inclusion of more complex algorithms like generalized additive models and polynomial splines.

DNA methylation and phenotypic data used to construct all predictors, as well as most testing datasets, is publicly available on the Gene Expression Omnibus (GEO), with the corresponding GEO accession numbers specified in **Table 1** [28].

**Table 1:** Characteristics of each included training and testing dataset.

Data Use	Data Source	DNA Source	N	Age Range (years)	Description
*Childhood Clock*					
Training	GSE32149	Child Blood	14	3.5-17.5	Subset of healthy control peripheral blood [29]
Training	GSE80283	Child Blood	183	-0.3--0.1	Dried blood spots taken for newborn screening [30]
Training	GSE79056	Cord Blood	36	-0.3-0.4	Cord blood samples from the Nashville Birth Cohort [31]
Training	GSE80261	Buccal	216	5 - 18	Buccal samples of FASD cases and controls [32]
Training	GSE50759	Buccal	41	1-21	Buccal samples from controls [33]
Training	GSE36054	Child Blood	134	1-16.9	Healthy children [34]
Training	GSE62924	Cord Blood	38	-0.1-0.04	Cord blood samples from the Biomarkers of Arsenic cohort [35]
Training	GSE73103	Child Blood	197	14.0-21.0	Healthy adolescent peripheral blood [36]
Testing	CHAMACOS	Cord/Child Blood	976	-0.1-15.1	CHAMACOS samples at 0, 7, 9, and 14 year timepoints [14,37,38]
Testing	CHAMACOS	Cord/Child Blood	386	-0.1-14.3	Duplicate samples measured with both 450K/EPIC [14]
Testing	GSE193879	Child Blood	127	0.1-17.0	COVID-19 cases and controls [39]
*Hannum Clock*					
Training	GSE40279	Peripheral Blood	656	19-101	Original Hannum Clock training dataset [3]
Testing	GSE55763	Peripheral Blood	22	43.0-74.6	Small subset of 11 duplicate pairs [40]
Testing	GSE84727	Peripheral Blood	665	18.3-80.7	Schizophrenia cases and controls [41]
Testing	GSE174422	Peripheral Blood	256	36.6-75.1	128 duplicate pairs from the Sister Study [42]
*PBB Predictor*					
Training	GSE116339	Peripheral Blood	505	23.0-88.5	Participants of the Michigan PBB registry (training split) [43]
Testing	GSE116339	Peripheral Blood	168	31.3-82.9	Participants of the Michigan PBB registry (testing split) [43]

#### Childhood clock.

Three novel epigenetic clocks were trained to predict chronological age in children using the three predictor development methods. All training data was obtained from the GEO under accession numbers GSE32149 [29], GSE80283 [30], GSE79056 [31], GSE80261 [32], GSE50759 [33], GSE36054 [34], GSE62924 [35], and GSE73103 [36]. The training dataset consisted of 859 samples of cord blood, child blood, and buccal cells obtained from children ranging from birth to 21 years of age, all measured with the 450K array. Each dataset was trimmed to 290207 CpG sites present across all training datasets, the primary CHAMACOS testing datasets, and still present on the EPIC/EPICv2 array manifests. To reflect variability in gestational age at birth, gestational age in cord blood samples was converted to age in years using the formula*: Age*_*Years*_ = 0 – ((39 – GA)/52.1429). A gestational age at birth of 39 weeks was used as a base age of 0. Age in years for all training samples was transformed using the formula: *Age*_*Transformed*_ = log(Age + 1) – log(21 + 1), based on the age transformation applied to pediatric samples for the training of the Horvath panTissue clock [2]. Full details of each training and testing dataset used are provided in **Table 1**. Training model and predictor specifications are provided in **Table A in** **S1 Text**. The resultant three clocks are referred to as i) the Childhood CpG clock, ii) the Childhood PCA clock, and iii) the Childhood SL PCA clock.

Several datasets were used to test the three childhood clocks. Firstly, 982 DNA methylation measurements derived from cord blood and child blood from 449 unique CHAMACOS birth cohort participants ranging in age from birth to 15.1 years old were used to test the three clocks in a longitudinal setting consisting of samples consisting of multiple DNA methylation arrays and DNA sources [14,37,38]. The 450K array was used to measure DNAm in cord blood at birth (n=372) and peripheral blood in the 9-year timepoint (n=240), and the EPIC array was used to measure DNAm in peripheral blood at the 7-year (n=183) and 14-year timepoints (n=187). Three samples from the birth timepoint and three samples from childhood timepoints were removed from analysis due to presenting as severe outliers across each of the three childhood clocks and the Horvath panTissue clock, resulting in a final sample size of 976. Second, an additional sample of 193 participants from the CHAMACOS birth cohort sample had duplicate DNA methylation measures obtained from the alternate array available to compare agreement of predictions between arrays [14]. At birth, 108 samples had matching 450K and EPIC measurements, and, at 14 years, 85 samples had matching 450K and EPIC measurements. Third, the GSE193879 public dataset was accessed from GEO, consisting of EPIC methylation measurements from 127 pediatric COVID-19 cases and controls ranging from 4 days old to 17 years old [39].

#### Hannum clock recreation.

The original dataset used to train the Hannum clock was obtained from GEO using accession number GSE40279, consisting of 656 adult whole blood samples ranging in age from 19 to 101 years old [3]. Three versions of the Hannum epigenetic clock were trained to predict chronological age using the development pipeline; the Hannum CpG clock, the Hannum PCA clock, and the Hannum SL PCA clock. The original Hannum clock additionally considered patient gender, BMI, diabetes status, ethnicity, and batch in their training models, however, because not all of these covariates were publicly available, our reconstructed Hannum clocks only considered DNA methylation for the training process. Each dataset was trimmed to 348546 CpG sites present in the Hannum training dataset and primary testing datasets. Several datasets were used to compare the Hannum clocks. First, the GSE55763 dataset consisting of duplicate DNA methylation measures from 11 adult participants was used to characterize agreement between duplicates [40]. Second, the GSE84727 dataset consisting of 665 DNA methylation measures from whole blood samples from schizophrenia cases and controls was used to characterize fit between chronological age, as well as ability to detect biologically-relevant associations [41]. Third, the GSE174422 dataset consisting of 256 DNA methylation measures from whole blood of 128 duplicate pairs from the Sister Study was used to characterize agreement between duplicates [42].

#### Polybrominated biphenyl (PBB) exposure predictor.

The GSE116339 dataset, consisting of 673 adults from the Michigan PBB registry with EPIC DNA methylation data and log-transformed total PBB measures available, was used to train three continuous predictors of log-transformed PBB exposure [43]. The total PBB measure was calculated as a summary measure of four individual PBB congeners: PBB-153, PBB-101, PBB-77, and PBB-180, measured in serum. Details on the Michigan PBB registry and PBB measures is detailed in Curtis et al. 2019 [43]. The dataset was randomly split into 505 training samples and 168 testing samples and trimmed to 348629 CpG sites present on both the PBB dataset and the 450K array. To examine the potential influence of differential training/testing data splits, we repeated this training process with 10 random training/testing data splits, calculated summary statistics for each permutation, and averaged correlation and MAE across all 10 permutations.

All final predictors generated for this study are accessible on Dryad (10.5061/dryad.p8cz8w9z3) [44]. For reference on the relative time requirements for training each predictor, the training process on our local computer (32GB RAM) for the childhood clock requires approximately 8.6 minutes (split up as ~57 seconds for CpG clock training, ~413 seconds for PCA, ~1 second for PCA clock training, and ~46 seconds for SL PCA clock training), training of the Hannum clock requires approximately 6.9 minutes (~59 seconds for CpG clock training, ~312 seconds for PCA, ~1 second for PCA clock training, and ~41 seconds for SL PCA clock training), and training of the PBB predictor requires approximately 4.7 minutes (~53 seconds for CpG clock training, ~190 seconds for PCA, ~1 second for PCA clock training, and ~38 seconds for SL PCA clock training). In all cases, PCA is the rate limiting step, however, PCA is only run once in order to train both the PCA and SL PCA clock.

### Statistical analysis

Person’s correlation coefficients and median absolute error (MAE) were calculated to characterize associations between epigenetic age and chronological age, as well as for agreement between duplicate samples. (**Table 2**) Furthermore, we used bootstrapping to assess differences in the fit for the predictions generated from each of the methods by resampling predictions with replacement, computing Pearson correlation coefficients and MAE between chronological age and predictions for each of the predictors, calculating the difference in correlation coefficients between each of the three methods, repeating the process with 10,000 iterations, and calculating the 95% confidence interval (CI) for the differences in correlation and MAE across all iterations.

**Table 2:** Correlation coefficients and median absolute error (MAE) for all analyses.

	CpG Predictor		PCA Predictor		SL PCA Predictor	
**Dataset**	**Corr.**	**MAE**	**Corr.**	**MAE**	**Corr.**	**MAE**
**Childhood Clock**						
Childhood Training Sample	0.943	0.815	0.962	0.663	0.965	0.660
CHAMACOS Full Sample	0.962	0.544	0.952	0.470	0.954	0.500
CHAMACOS Cord Blood	0.135	0.248	0.342	0.095	0.339	0.105
CHAMACOS Childhood	0.756	1.444	0.691	1.345	0.702	1.388
CHAMACOS Duplicates*	0.997	0.110	0.998	0.111	0.998	0.108
GSE193879 Testing Sample	0.914	1.122	0.892	1.875	0.895	1.861
**Hannum Clock**						
Hannum Training Sample	0.970	2.572	0.952	3.325	0.983	2.255
GSE84727 Schizophrenia Testing Sample	0.972	3.240	0.932	5.064	0.946	5.121
GSE55763 Duplicates*	0.989	1.250	0.997	0.285	0.997	0.422
GSE174422 Duplicates*	0.983	0.902	0.985	0.999	0.981	1.108
**PBB Predictor**						
Training Sample	0.804	0.622	0.482	0.766	0.631	0.725
Testing Sample	0.372	0.736	0.325	0.730	0.335	0.672

Correlation and MAE calculated against observed chronological age for most samples, and between predictions from replicate measures for duplicate samples denoted with a “*”.

Furthermore, to benchmark the performance of our predictors, we calculated 5 commonly used epigenetic clocks (Horvath pan-Tissue, PC Horvath, PhenoAge, PC PhenoAge, and PC GrimAge) using the R *methscore* function with fast imputation and normalization set to true [45]. We include the PC GrimAge clock to compare the applicability of our clocks to several type of analysis, however, we note that the PC GrimAge predictor includes chronological age as a predictor, limiting the ability to directly evaluate its performance as a predictor of chronological age. Additional analyses were conducted to test the applicability of the childhood clocks towards a longitudinal analysis in the CHAMACOS cohort. Building off of a previous cross-sectional analysis within the CHAMACOS cohort [46], generalized estimating equations (GEE) were used to model the associations between log2 transformed pregnancy-averaged measures of phthalate metabolite exposure and cell-adjusted epigenetic age acceleration measures, further adjusted for maternal poverty category (at or below poverty, poverty - 200%, >200% poverty), parity (0, 1, 2+), maternal age (continuous), maternal smoking (yes or no), and maternal BMI (continuous). Cell-adjusted epigenetic age acceleration measures were calculated by extracting the residuals from a regression of each childhood epigenetic age estimate on chronological age, further adjusted for cell proportions (CD8T, CD4T, NK, Granulocyte, Monocyte, and B cell) calculated using the *EpiDISH* method [47]. GEE models were run using a subset of 112 participants with DNA methylation data available at all four timepoints. Both overall models further adjusted for child sex and sex-stratified models were run. Additional details on the phthalate, DNA methylation, and covariate measurement ascertainment are provided in Khodasevich et al 2023 [46].

For testing of the associations between schizophrenia case status and epigenetic age acceleration, epigenetic age acceleration measures were calculated as the residuals from a regression of each epigenetic age estimate on chronological age. General linear regression models were then run with each clock’s epigenetic age acceleration measure as the outcome and schizophrenia case status as the exposure, further adjusted for patient sex.

## Supporting information

S1 Text Table A. Training model specifications for each predictor. Specifications denote the SuperLearner library used for training, the number of low variance principal components that were trimmed prior to training, training dataset size, and age transformation. Details on number of CpGs and PCs selected in the CpG and PCA predictors respectively, as well as weights given to each algorithm for the SL PCA predictor are provided. SL.glmnetXX parameters refer to glmnet models run with the alpha parameter set to 0.XX. Table B. Correlation coefficients and median absolute error (MAE) for the Horvath, PC Horvath, PhenoAge, PC PhenoAge, and PC GrimAge clock for each of the chronological age testing datasets. Correlation and MAE calculated against observed chronological age for most samples, and between predictions from replicate measures for duplicate samples denoted with a “*”. Fig A. Additional childhood clock testing. Correlations and median absolute error (MAE) of each clock’s epigenetic age prediction in cord blood with actual gestational age at birth (a–c) and with age in childhood (d–f) in the CHAMACOS cohort. Correlation coefficients and median absolute error (MAE) to chronological age for each childhood clock development method for the GSE193879 testing data (g–i). The 1:1 line is shown in black. Fig B. CHAMACOS longitudinal testing model summaries. Beta coefficients and 95% confidence intervals from the generalized estimating equation models for associations between pregnancy-average phthalate measures and cell-adjusted epigenetic age acceleration. Overall models and sex-stratified models are presented. Fig C. CHAMACOS longitudinal testing. Box plots displaying the distribution of 95% confidence interval widths derived from the three clocks from the generalized estimating equation models for associations between pregnancy-average phthalate measures and cell-adjusted epigenetic age acceleration. Fig D. CHAMACOS longitudinal testing model summaries for the external epigenetic clocks. Beta coefficients and 95% confidence intervals from the generalized estimating equation models for associations between pregnancy-average phthalate measures and cell-adjusted epigenetic age acceleration. Overall models and sex-stratified models are presented. Fig E. Boxplots displaying the distribution of 95% confidence interval widths derived from the external epigenetic clocks from the generalized estimating equation models for associations between pregnancy-average phthalate measures and cell-adjusted epigenetic age acceleration. Fig F. Correlation coefficients and median absolute error (MAE) to chronological age for each Hannum clock development method for the GSE174422 Sister Study testing data. Each set of duplicate samples are presented separately in (a–c) and (d–f). The agreement between duplicate samples is shown in (g–i). The 1:1 line is shown in black. Fig G. Schematic detailing the overall flow for the SL PCA training pipeline.
(DOCX)
