# Supplementary material for: A SuperLearner-based pipeline for the development of DNA methylation-derived predictors of phenotypic traits
Source: PLoS Comput Biol. 2025 Feb 6;21(2):e1012768. doi: 10.1371/journal.pcbi.1012768 (PMC11801726; doi:10.1371/journal.pcbi.1012768)
Supplement: S1 Text — Table A. Training model specifications for each predictor. Specifications denote the SuperLearner library used for training, the number of low variance principal components that were trimmed prior to training, training dataset size, and age transformation. Details on number of CpGs and PCs selected in the CpG and PCA predictors respectively, as well as weights given to each algorithm for the SL PCA predictor are provided. SL.glmnetXX parameters refer to glmnet models run with the alpha parameter set to 0.XX. Table B. Correlation coefficients and median absolute error (MAE) for the Horvath, PC Horvath, PhenoAge, PC PhenoAge, and PC GrimAge clock for each of the chronological age testing datasets. Correlation and MAE calculated against observed chronological age for most samples, and between predictions from replicate measures for duplicate samples denoted with a “*”. Fig A. Additional childhood clock testing. Correlations and median absolute error (MAE) of each clock’s epigenetic age prediction in cord blood with actual gestational age at birth (a–c) and with age in childhood (d–f) in the CHAMACOS cohort. Correlation coefficients and median absolute error (MAE) to chronological age for each childhood clock development method for the GSE193879 testing data (g–i). The 1:1 line is shown in black. Fig B. CHAMACOS longitudinal testing model summaries. Beta coefficients and 95% confidence intervals from the generalized estimating equation models for associations between pregnancy-average phthalate measures and cell-adjusted epigenetic age acceleration. Overall models and sex-stratified models are presented. Fig C. CHAMACOS longitudinal testing. Box plots displaying the distribution of 95% confidence interval widths derived from the three clocks from the generalized estimating equation models for associations between pregnancy-average phthalate measures and cell-adjusted epigenetic age acceleration. Fig D. CHAMACOS longitudinal testing model summaries for the external epige [file pcbi.1012768.s001.docx]

**A SuperLearner-based Pipeline for the Development of DNA Methylation-derived Predictors of Phenotypic Traits**

Dennis Khodasevich ^a^, Nina Holland ^b^, Lars van der Laan ^c^, Andres Cardenas ^a*^

**Supplemental Information File 1**

*^a^ Department of Epidemiology and Population Health, Stanford University School of Medicine, Palo Alto, California, United States of America*

*^b^ Center for Environmental Research and Community Health (CERCH), University of California Berkeley School of Public Health, Berkeley, California, United States of America*

*^c^ Department of Statistics, University of Washington, Seattle, Washington, United States of America*

******* *andresca@stanford.edu*

**List of Legends in the Supporting Information Document

*Table A in S1 Text****: Training model specifications for each predictor. Specifications denote the SuperLearner library used for training, the number of low variance principal components that were trimmed prior to training, training dataset size, and age transformation. Details on number of CpGs and PCs selected in the CpG and PCA predictors respectively, as well as weights given to each algorithm for the SL PCA predictor are provided. SL.glmnetXX parameters refer to glmnet models run with the alpha parameter set to 0.xx****Table B in S1 Text****: Correlation coefficients and median absolute error (MAE) for the Horvath, PC Horvath, PhenoAge, PC PhenoAge, and PC GrimAge clock for each of the chronological age testing datasets. Correlation and MAE calculated against observed chronological age for most samples, and between predictions from replicate measures for duplicate samples denoted with a “*”****Figure A in S1 Text****: Additional childhood clock testing. Correlations and median absolute error (MAE) of each clock’s epigenetic age prediction in cord blood with actual gestational age at birth (a-c) and with age in childhood (d-f) in the CHAMACOS cohort. Correlation coefficients and median absolute error (MAE) to chronological age for each childhood clock development method for the GSE193879 testing data (g-i). The 1:1 line is shown in black.****Figure B in S1 Text****: CHAMACOS longitudinal testing model summaries. Beta coefficients and 95% confidence intervals from the generalized estimating equation models for associations between pregnancy-average phthalate measures and cell-adjusted epigenetic age acceleration. Overall models and sex-stratified models are presented.****Figure C in S1 Text****: CHAMACOS longitudinal testing. Box plots displaying the distribution of 95% confidence interval widths derived from the three clocks from the generalized estimating equation models for associations between pregnancy-average phthalate measures and cell-adjusted epigenetic age acceleration.****Figure D in S1 Text****: CHAMACOS longitudinal testing model summaries for the external epigenetic clocks. Beta coefficients and 95% confidence intervals from the generalized estimating equation models for associations between pregnancy-average phthalate measures and cell-adjusted epigenetic age acceleration. Overall models and sex-stratified models are presented.****Figure E in S1 Text****: Boxplots displaying the distribution of 95% confidence interval widths derived from the external epigenetic clocks from the generalized estimating equation models for associations between pregnancy-average phthalate measures and cell-adjusted epigenetic age acceleration.*

***Figure F in S1 Text****: Correlation coefficients and median absolute error (MAE) to chronological age for each Hannum clock development method for the GSE174422 Sister Study testing data. Each set of duplicate samples are presented separately in (a-c) and (d-f). The agreement between duplicate samples is shown in (g-i). The 1:1 line is shown in black.****Figure G in S1 Text****: Schematic detailing the overall flow for the SL PCA training pipeline.*

| **Predictor** | **Specifications** |
| --- | --- |
| **Childhood Clock** |  |
| SuperLearner Library | "SL.mean", c("SL.glm", "screen.glmnet"), "SL.glm", "SL.glmnet0", "SL.glmnet05", "SL.glmnet10", "SL.glmnet25", "SL.glmnet40", "SL.glmnet50", "SL.glmnet60", "SL.glmnet75", "SL.glmnet90" |
| Trim Length | 15 |
| Training Sample Size | 859 |
| Number of CpGs | 290207 |
| Age Transformation | Horvath |
| CpG Clock Components | 42 |
| PCA Clock Components | 131 |
| SuperLearner Weights | SL.glmnet25_All: 0.5563497, SL.glmnet10_All: 0.3791394, SL.glm_screen.glmnet: 0.06448999, SL.glm_All: 2.092854e-05 |
|  |  |
| **Hannum Clock** |  |
| SuperLearner Library | "SL.mean", c("SL.glm", "screen.glmnet"), "SL.glm", "SL.glmnet0", "SL.glmnet05", "SL.glmnet10", "SL.glmnet25", "SL.glmnet40", "SL.glmnet50", "SL.glmnet60", "SL.glmnet75", "SL.glmnet90" |
| Trim Length | 15 |
| Training Sample Size | 656 |
| Number of CpGs | 348546 |
| Age Transformation | None |
| CpG Clock Components | 299 |
| PCA Clock Components | 137 |
| SuperLearner Weights | SL.glmnet10_All: 0.6420032453, SL.glmnet25_All: 0.3578708535, SL.glm_All: 0.0001259012 |
|  |  |
| **PBB Predictor** |  |
| SuperLearner Library | "SL.mean", c("SL.gam", "screen.glmnet"), c("SL.polymars", "screen.glmnet"), "SL.glmnet0", "SL.glmnet05", "SL.glmnet10", "SL.glmnet25", "SL.glmnet40", "SL.glmnet50", "SL.glmnet60", "SL.glmnet75", "SL.glmnet90" |
| Trim Length | 1 |
| Training Sample Size | 505 |
| Number of CpGs | 348629 |
| Age Transformation | None |
| CpG Clock Components | 135 |
| PCA Clock Components | 20 |
| SuperLearner Weights | SL.glmnet40_All: 0.7185691, SL.gam_screen.glmnet: 0.1506592, SL.polymars_screen.glmnet: 0.1307717 |

***Table A****: Training model specifications for each predictor. Specifications denote the SuperLearner library used for training, the number of low variance principal components that were trimmed prior to training, training dataset size, and age transformation. Details on number of CpGs and PCs selected in the CpG and PCA predictors respectively, as well as weights given to each algorithm for the SL PCA predictor are provided. SL.glmnetXX parameters refer to glmnet models run with the alpha parameter set to 0.xx*

|  | Horvath | PC Horvath | PhenoAge | PC  PhenoAge | PC  GrimAge^ |
| --- | --- | --- | --- | --- | --- |
| **Childhood Clock** |  |  |  |  |  |
| CHAMACOS Full Sample | 0.970 (2.579) | 0.978 (7.304) | 0.930 (7.637) | 0.953 (5.827) | 0.858 (22.882) |
| CHAMACOS Cord Blood | 0.108 (1.445) | 0.212 (1.746) | 0.212 (19.645) | 0.243 (17.532) | 0.210 (25.813) |
| CHAMACOS Childhood | 0.841 (4.078) | 0.873 (9.538) | 0.668 (3.879) | 0.766 (3.054) | 0.793 (21.382) |
| CHAMACOS Duplicates* | 0.989 (0.360) | 0.998 (0.228) | 0.987 (2.475) | 0.997 (2.755) | 0.993 (0.650) |
| GSE193879 Testing Sample | 0.886 (3.886) | 0.935 (11.521) | 0.767 (4.867) | 0.831 (5.569) | 0.863 (23.034) |
|  |  |  |  |  |  |
| **Hannum Clock** |  |  |  |  |  |
| GSE84727 Testing Sample | 0.938 (7.206) | 0.936 (8.275) | 0.910 (3.914) | 0.930 (3.254) | 0.931 (15.483) |
| GSE55763 Duplicates* | 0.937 (2.135) | 0.990 (0.334) | 0.919 (3.012) | 0.998 (0.453) | 0.999 (0.181) |
| GSE174422 Duplicates* | 0.957 (1.704) | 0.983 (0.894) | 0.946 (2.422) | 0.983 (0.578) | 0.997 (0.431) |

***Table B****: Correlation coefficients and median absolute error (MAE) for the Horvath, PC Horvath, PhenoAge, PC PhenoAge, and PC GrimAge clock for each of the chronological age testing datasets. Correlation and MAE calculated against observed chronological age for most samples, and between predictions from replicate measures for duplicate samples denoted with a “*”
^ The PC GrimAge clock contains chronological age as a predictor, limiting the ability to directly evaluate its performance as a predictor of age.*

**
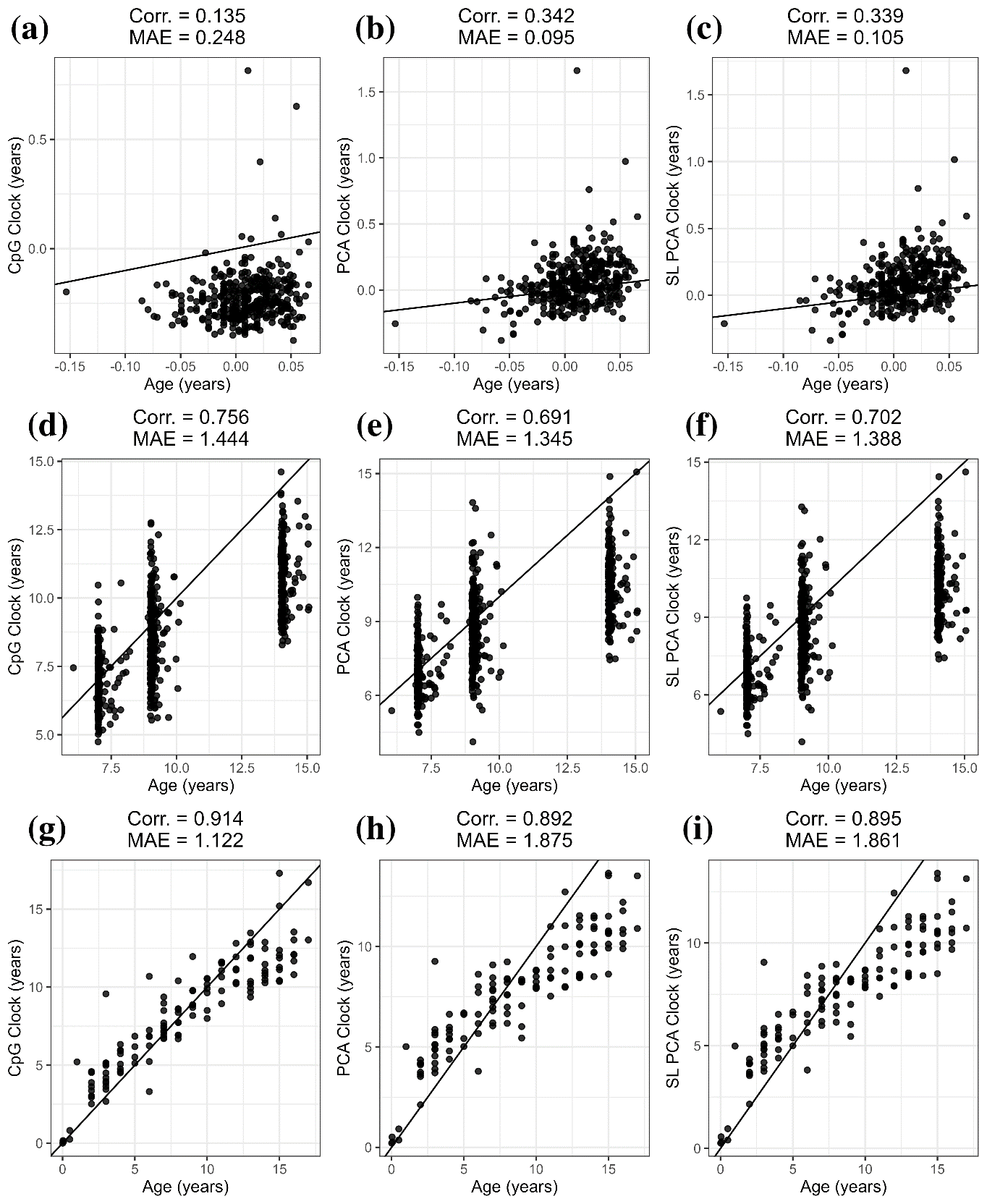
**

***Figure A****: Additional childhood clock testing. Correlations and median absolute error (MAE) of each clock’s epigenetic age prediction in cord blood with actual gestational age at birth (a-c) and with age in childhood (d-f) in the CHAMACOS cohort. Correlation coefficients and median absolute error (MAE) to chronological age for each childhood clock development method for the GSE193879 testing data (g-i). The 1:1 line is shown in black.*

**
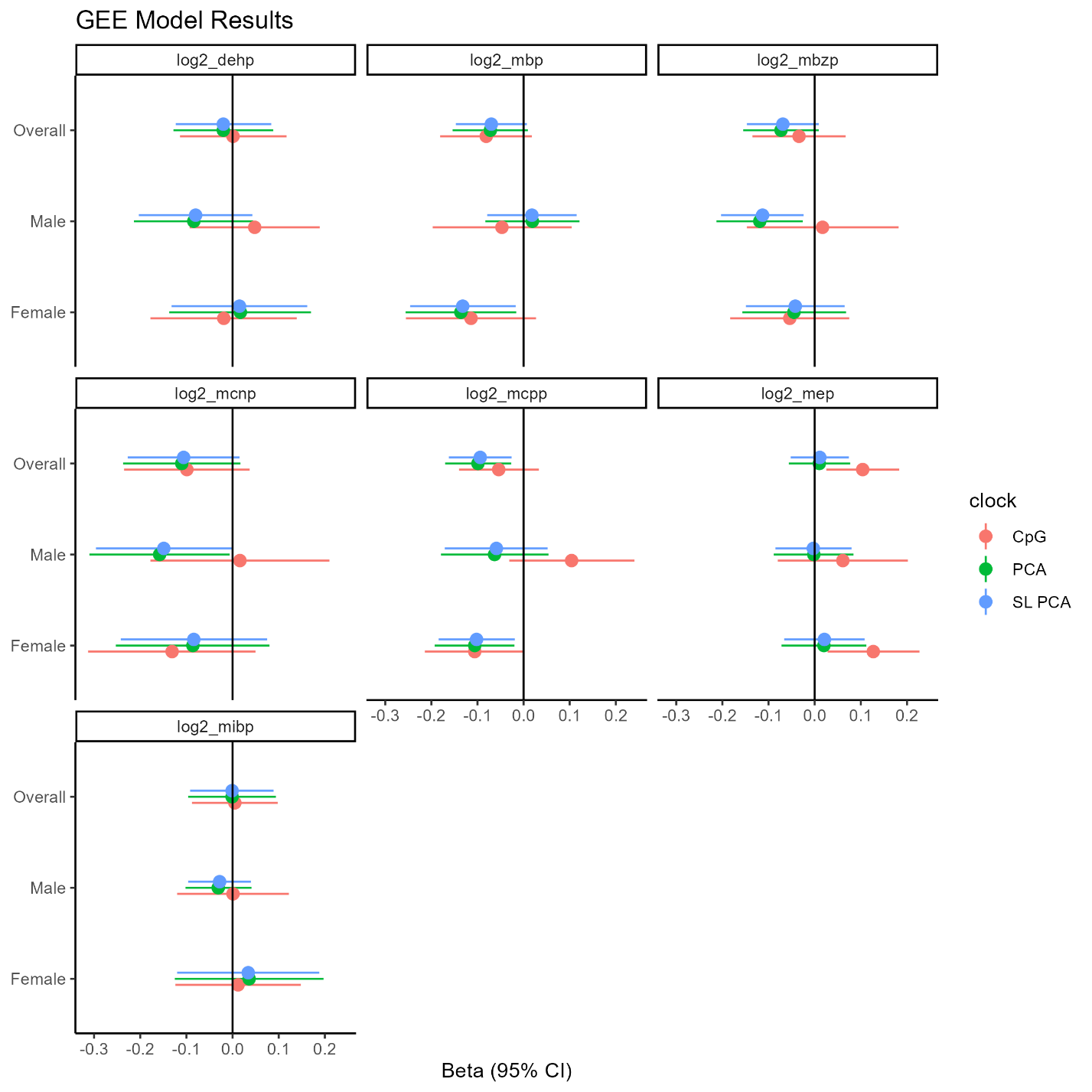
**

***Figure B****: CHAMACOS longitudinal testing model summaries. Beta coefficients and 95% confidence intervals from the generalized estimating equation models for associations between pregnancy-average phthalate measures and cell-adjusted epigenetic age acceleration. Overall models and sex-stratified models are presented.*

**
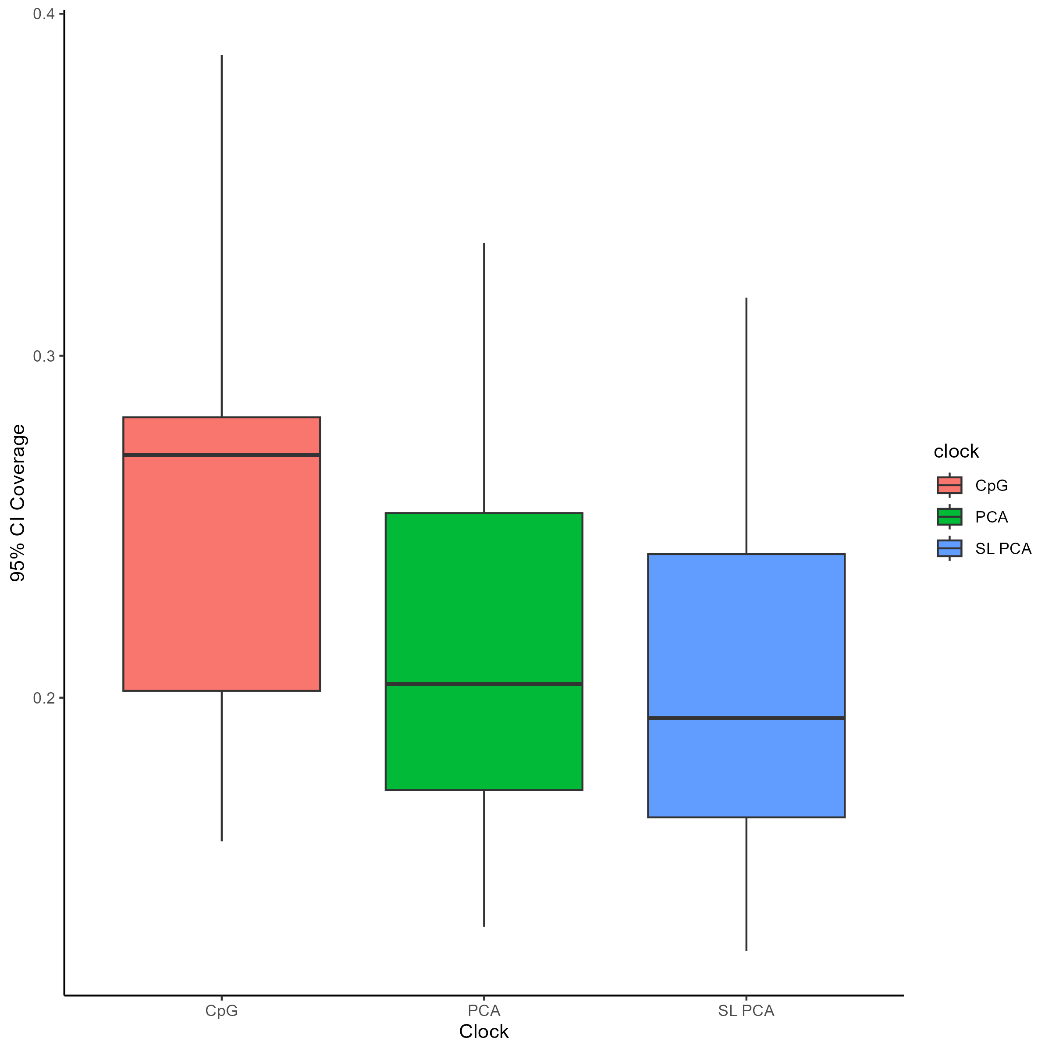
**

***Figure C****: CHAMACOS longitudinal testing. Box plots displaying the distribution of 95% confidence interval widths derived from the three clocks from the generalized estimating equation models for associations between pregnancy-average phthalate measures and cell-adjusted epigenetic age acceleration.*

**
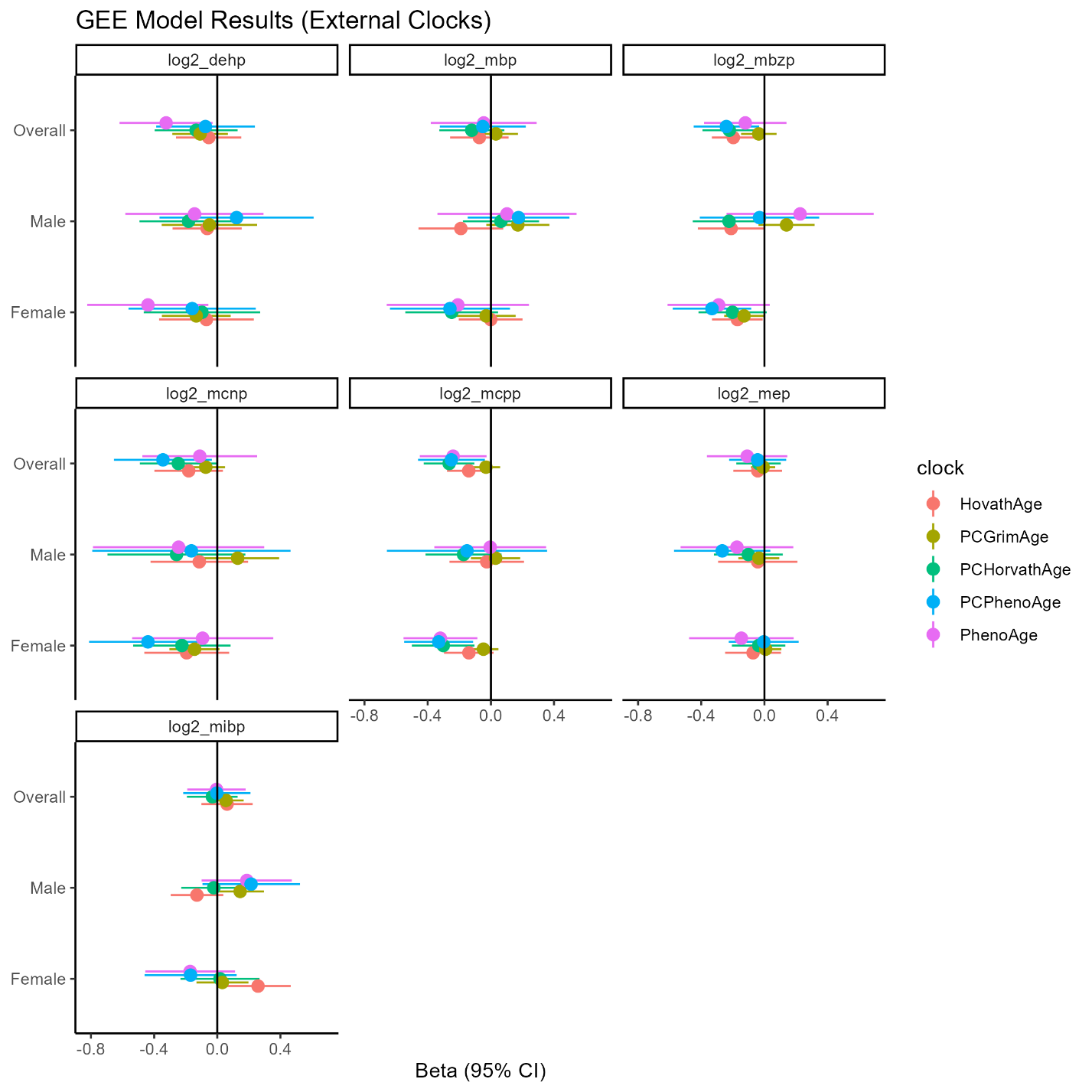
**

***Figure D****: CHAMACOS longitudinal testing model summaries for the external epigenetic clocks. Beta coefficients and 95% confidence intervals from the generalized estimating equation models for associations between pregnancy-average phthalate measures and cell-adjusted epigenetic age acceleration. Overall models and sex-stratified models are presented.*

**
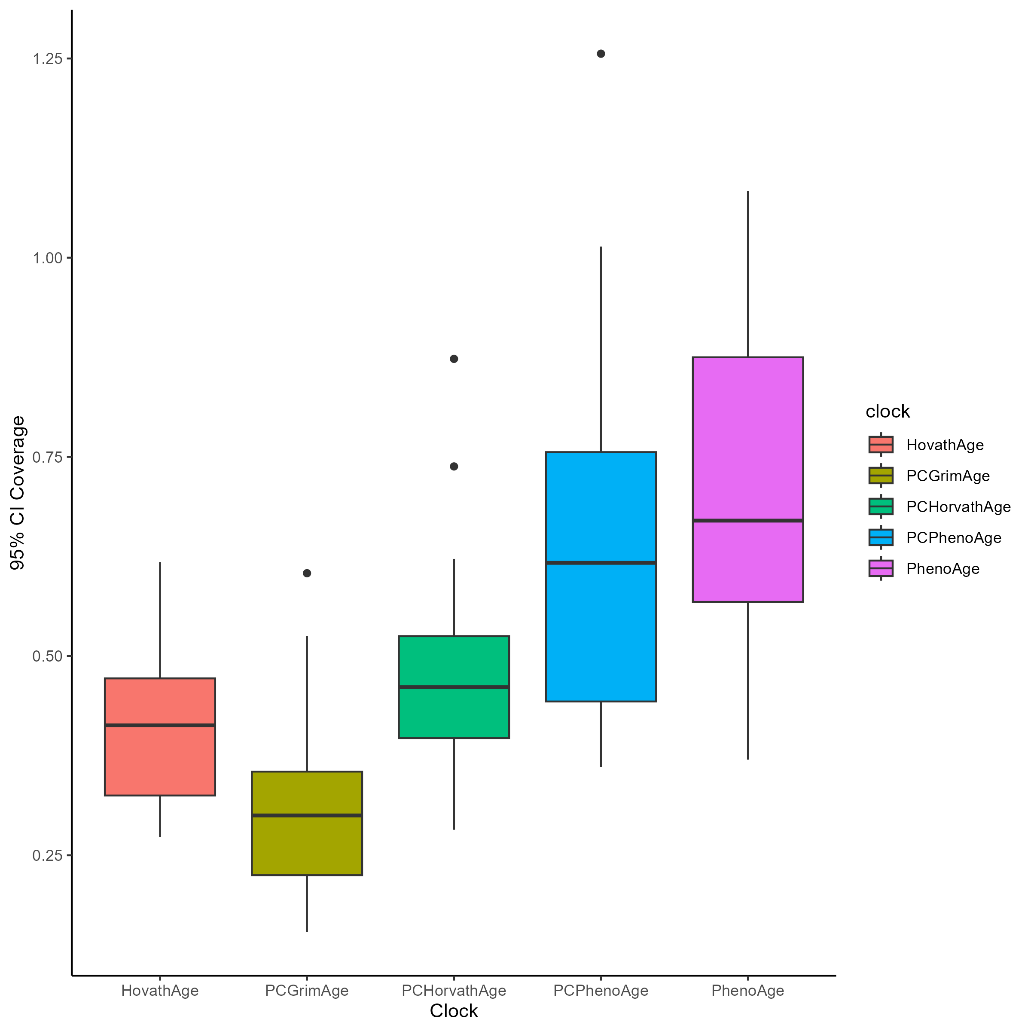
**

***Figure E****: Boxplots displaying the distribution of 95% confidence interval widths derived from the external epigenetic clocks from the generalized estimating equation models for associations between pregnancy-average phthalate measures and cell-adjusted epigenetic age acceleration.*

**
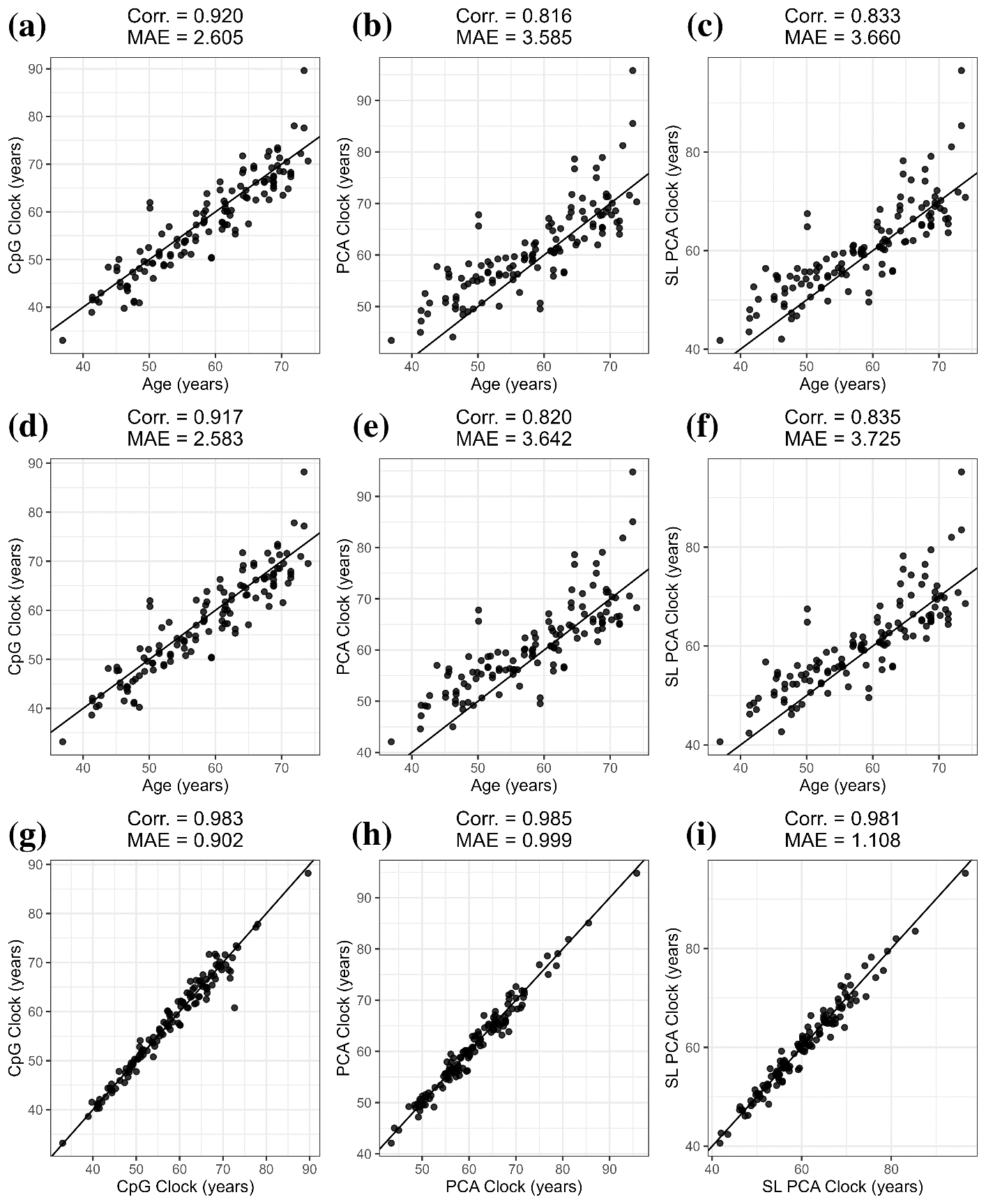
**

***Figure F****: Correlation coefficients and median absolute error (MAE) to chronological age for each Hannum clock development method for the GSE174422 Sister Study testing data. Each set of duplicate samples are presented separately in (a-c) and (d-f). The agreement between duplicate samples is shown in (g-i). The 1:1 line is shown in black.*

*
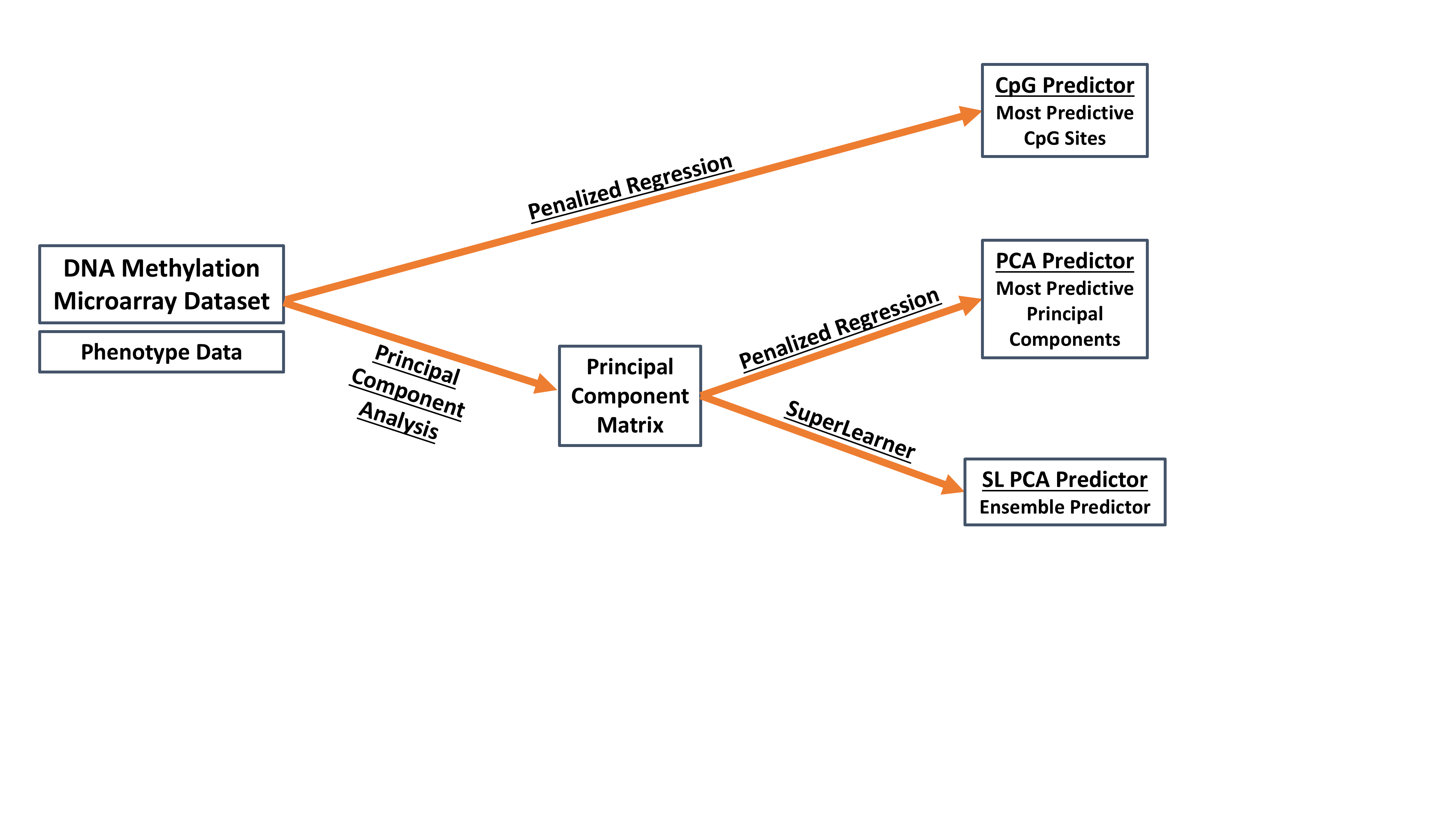
*

***Figure G****: Schematic detailing the overall flow for the SL PCA training pipeline.*
